# Supplementary material for: Different Approaches to Appraising Systematic Reviews of Digital Interventions for Physical Activity Promotion Using AMSTAR 2 Tool: Cross-Sectional Study
Source: Int J Environ Res Public Health. 2023 Mar 7;20(6):4689. doi: 10.3390/ijerph20064689 (PMC10048476; doi:10.3390/ijerph20064689)
Supplement: Supplementary file 1 [file ijerph-20-04689-s001.zip › DigitEVA_AMSTAR2_Supplementary_Materials_Table_S1.pdf]

Supplementary Materials Table S1

De Santis, K.; Matthias, K. Different approaches to appraising systematic reviews of digital interventions for physical activity promotion using AMSTAR 2 tool: Cross-sectional study. *Int. J. Environ. Res. Public Health* **2023**, https://doi.org/10.3390ijerph20064689

Table S1. STROBE Checklist

Item	Item No	Recommendation	Page No.
**Title and abstract**	1	(*a*) Indicate the study's design with a commonly used term in the title or the abstract	1
		(*b*) Provide in the abstract an informative and balanced summary of what was done and what was found	1
**Introduction**			
Background/ rationale	2	Explain the scientific background and rationale for the investigation being reported	1-2
Objectives	3	State specific objectives, including any prespecified hypotheses	2
**Methods**			
Study design	4	Present key elements of study design early in the paper	Section 2.2
Setting	5	Describe the setting, locations, and relevant dates, including periods of recruitment, exposure, follow-up, and data collection	Section 2.2
Participants	6	(*a*) *Cross-sectional study*—Give the eligibility criteria, and the sources and methods of selection of participants	Section 2.3
Variables	7	Clearly define all outcomes, exposures, predictors, potential confounders, and effect modifiers. Give diagnostic criteria, if applicable	Section 2.5
Data sources/ measuremen t	8*	For each variable of interest, give sources of data and details of methods of assessment (measurement). Describe comparability of assessment methods if there is more than one group	Section 2.4
Bias	9	Describe any efforts to address potential sources of bias	Sections 2.3-2.4
Study size	10	Explain how the study size was arrived at	Section 2.3
Quantitative variables	11	Explain how quantitative variables were handled in the analyses. If applicable, describe which groupings were chosen and why	Sections 2.5-2.6
Statistical methods	12	(*a*) Describe all statistical methods, including those used to control for confounding	Section 2.6
		(*b*) Describe any methods used to examine subgroups and interactions	Section 2.6
		(*c*) Explain how missing data were addressed	-
		(*d*) *Cross-sectional study*—If applicable, describe analytical methods taking account of sampling strategy	-

		(*e*) Describe any sensitivity analyses	Section 2.6
**Results**			**Page No.**
Participants	13*	(a) Report numbers of individuals at each stage of study—eg numbers potentially eligible, examined for eligibility, confirmed eligible, included in the study, completing follow-up, and analysed	Section 3.1
		(b) Give reasons for non-participation at each stage	-
		(c) Consider use of a flow diagram	-
Descriptive data	14*	(a) Give characteristics of study participants (eg demographic, clinical, social) and information on exposures and potential confounders	Section 3.1
		(b) Indicate number of participants with missing data for each variable of interest	-
		(c) *Cohort study*—Summarise follow-up time (eg, average and total amount)	-
Outcome data	15*	*Cohort study*—Report numbers of outcome events or summary measures over time	-
		Case-control study—Report numbers in each exposure category, or summary measures of exposure	-
		Cross-sectional study—Report numbers of outcome events or summary measures	Sections 3.2-3.3
Main results	16	(*a*) Give unadjusted estimates and, if applicable, confounder-adjusted estimates and their precision (eg, 95% confidence interval). Make clear which confounders were adjusted for and why they were included	Section 3.2-3.3, Table 2, Figure 1
		(*b*) Report category boundaries when continuous variables were categorized	-
		(*c*) If relevant, consider translating estimates of relative risk into absolute risk for a meaningful time period	-
Other analyses	17	Report other analyses done—eg analyses of subgroups and interactions, and sensitivity analyses	Section 3.4, Table 3
**Discussion**			
Key results	18	Summarise key results with reference to study objectives	7-9
Limitations	19	Discuss limitations of the study, taking into account sources of potential bias or imprecision. Discuss both direction and magnitude of any potential bias	9
Interpretation	20	Give a cautious overall interpretation of results considering objectives, limitations, multiplicity of analyses, results from similar studies, and other relevant evidence	7-9
Generalisability	21	Discuss the generalisability (external validity) of the study results	9
**Other information**			

Funding	22	Give the source of funding and the role of the funders for the present study and, if applicable, for the original study on which the present article is based	9

Source: von Elm, E.; Altman, D.G.; Egger, M.; Pocock, S.J.; Gøtzsche, P.C.; Vandenbroucke, J.P.; for the, S.I. The Strengthening the Reporting of Observational Studies in Epidemiology (STROBE) Statement: Guidelines for Reporting Observational Studies. *PLoS Medicine* **2007**, *4*, e296, doi:10.1371/journal.pmed.0040296.

*Give information separately for cases and controls in case-control studies and, if applicable, for exposed and unexposed groups in cohort and cross-sectional studies.
